# Transcriptome Analysis of Kiwifruit (*Actinidia chinensis*) Bark in Response to Armoured Scale Insect (*Hemiberlesia lataniae*) Feeding

**DOI:** 10.1371/journal.pone.0141664

**Published:** 2015-11-16

**Authors:** M. Garry Hill, Kirstin V. Wurms, Marcus W. Davy, Elaine Gould, Andrew Allan, Nicola A. Mauchline, Zhiwei Luo, Annette Ah Chee, Kate Stannard, Roy D. Storey, Erik H. Rikkerink

**Affiliations:** 1 The New Zealand Institute for Plant & Food Research Limited (PFR), 412 No1 Rd RD2, Te Puke, New Zealand; 2 Plant & Food Research Limited (PFR), Private Bag 3230, Waikato Mail Centre, Hamilton, New Zealand; 3 Plant & Food Research Limited (PFR), Private Bag 92169, Auckland, New Zealand; Virginia Tech, UNITED STATES

## Abstract

The kiwifruit cultivar *Actinidia chinensis* ‘Hort16A’ is resistant to the polyphagous armoured scale insect pest *Hemiberlesia lataniae* (Hemiptera: Diaspididae). A cDNA microarray consisting of 17,512 unigenes selected from over 132,000 expressed sequence tags (ESTs) was used to measure the transcriptomic profile of the *A*. *chinensis* ‘Hort16A’ canes in response to a controlled infestation of *H*. *lataniae*. After 2 days, 272 transcripts were differentially expressed. After 7 days, 5,284 (30%) transcripts were differentially expressed. The transcripts were grouped into 22 major functional categories using MapMan software. After 7 days, transcripts associated with photosynthesis (photosystem II) were significantly down-regulated, while those associated with secondary metabolism were significantly up-regulated. A total of 643 transcripts associated with response to stress were differentially expressed. This included biotic stress-related transcripts orthologous with pathogenesis related proteins, the phenylpropanoid pathway, NBS-LRR (R) genes, and receptor-like kinase–leucine rich repeat signalling proteins. While transcriptional studies are not conclusive in their own right, results were suggestive of a defence response involving both ETI and PTI, with predominance of the SA signalling pathway. Exogenous application of an SA-mimic decreased *H*. *lataniae* growth on *A*. *chinensis* ‘Hort16A’ plants in two laboratory experiments.

## Introduction

**Fig 1:**
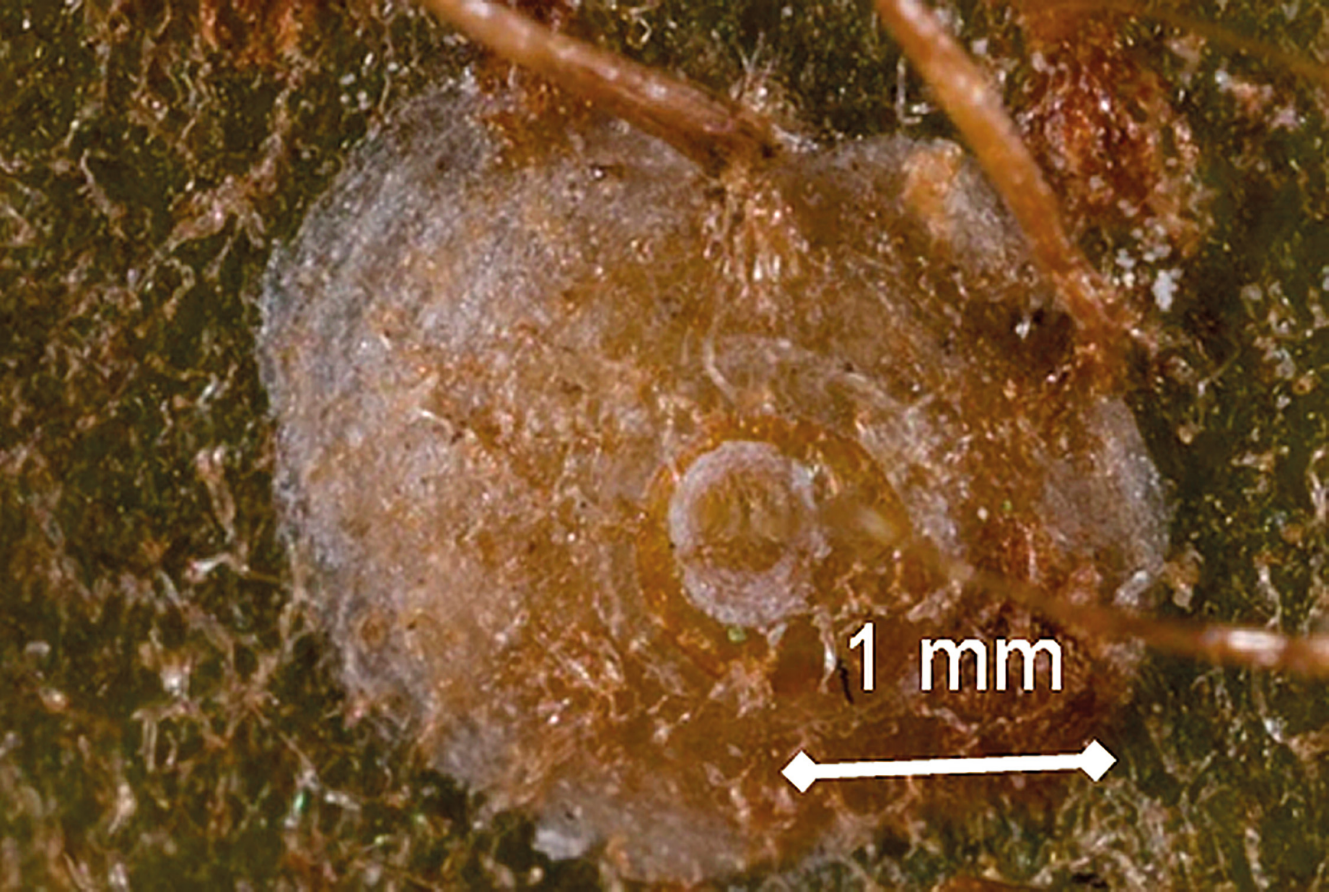
Adult *Hemiberlesia lataniae* armoured scale on the surface of an *Actinidia chinensis* ‘Hort16A’ fruit. The body is covered by a protective cap constructed of exuviae and waxy secretions.

Kiwifruit (genus *Actinidia*) has been grown commercially for over 40 years, mainly in New Zealand, Chile, Italy and China, where it has developed into an important horticultural crop [1]. In New Zealand, the three commercially grown species, the green-fleshed *A*. *deliciosa* (A. Chev.) C.F. Liang et A.R. Ferguson, the yellow-fleshed *A*. *chinensis* Planch. and the kiwiberry *A*. *arguta* (Sieb. et Zucc.) are attacked by a range of native and introduced pests [2]. Two introduced armoured scale insect species, latania scale (*Hemiberlesia lataniae* Signoret) and greedy scale (*H*. *rapax* Comstock) (Hemiptera: Diaspididae) are important pests of export kiwifruit crops. *H*. *lataniae* (Fig 1) was first recorded in New Zealand in the 1980s [3] and has spread to most kiwifruit-growing regions over the last 30 years [4], displacing greedy scale as the dominant species on the *A*. *deliciosa* ‘Hayward’ variety, but not on *A*. *chinensis* ‘Hort16A’ variety [4,5].

At the time that the commercial variety ‘Hort16A’ was bred (mid 1990s), nothing was known about the resistance of kiwifruit (*Actinidia* species) to insects, and the resistance of ‘Hort16A’ to *H*. *lataniae* is therefore serendipitous. Studies carried out on the susceptibility of a range of kiwifruit germplasm to insects in recent years has demonstrated considerable variation in susceptibility to armoured scale insects [6], highlighting the need to elucidate the genetic basis of kiwifruit resistance to these pests so that a range of useful and durable resistances can be incorporated into new varieties.

Plant immune responses to insect pests have been less intensely studied than responses to pathogens, and studies on chewing insects (Lepidoptera and Coleoptera) predominate over those on sap-sucking insects [7,8]. Plants possess two broad categories of immunity against pests and pathogens; pathogen (or herbivore) associated molecular pattern-triggered immunity (PTI (or HTI)) and effector-triggered immunity (ETI) [9]. In addition, insect feeding may induce a generalised wound response by the plant which will increase its resistance to insects. PTI is considered to be more general or ‘basal’ and evolutionarily ancient, while ETI tends to be specific and triggered by the recognition of specific effector (herbivore or pathogen) proteins by plant proteins associated with resistance (R) genes, many of which are characterised by conserved nucleotide binding site, leucine rich repeat (NBS-LRR) motifs. Growing evidence suggests that PTI and ETI may represent parts of a defence response continuum rather than being distinct mechanisms [10]. A recent study to sequence the *A*. *chinensis* ‘Hongyang’ genome [11] found relatively few (c.f. *Arabidopsis thaliana*, rice, grape and tomato) R-genes (96), and relatively large numbers (261) of putative pattern-recognition receptor (PRR) genes encoding receptor-like kinases (RLK-LRR). This led Huang et al. [11] to speculate that PTI may be the more important defensive response in kiwifruit than ETI.

*H*. *lataniae* females (only uniparental populations exist in New Zealand) are sessile for all but the first few hours of their life. The mobile first instar (crawler) stages usually begin settlement and scale cap construction within hours of emergence [12,13]. They feed by inserting their stylet (mandibles) intracellularly into parenchyma and collenchyma cells and emptying the contents, probably assisted by a salivary secretion [14]. Once inserted, the stylet remains permanently inside the plant and the insect must re-insert its stylet after each of its two moults (diaspids are neotenous and have only three life stages). *H*. *lataniae* is highly polyphagous, having been recorded feeding on species from 106 plant families [15]. Microscopy and histology studies of the interaction between *H*. *lataniae* and the commercial kiwifruit variety *A*. *chinensis* ‘Hort16A’ have shown that when the insect settles on the bark of this variety, the plant mounts a strong defence response, leading to wound periderm formation around the insect, localised cell death (hypersensitivity), phenolic deposition and death of the insect within 3–4 weeks of its settlement [14]. After 15 years of commercial use throughout several hundreds of hectares of commercial plantings in New Zealand, Italy and France, there are no reported instances of the breaking of the resistance of this highly successful commercial kiwifruit variety to *H*. *lataniae*. However, *A*. *chinensis* ‘Hort16A’ is now being phased out of production because of its susceptibility to bacterial canker disease (Psa), and there is an ongoing need to incorporate durable pest and disease resistance into new varieties. The microarray experiment reported here was undertaken to initiate studies into kiwifruit *A*. *chinensis* ‘Hort16A’ transcriptional changes associated with *H*. *lataniae* feeding with a view to assisting the development of pest-resistant or tolerant kiwifruit varieties. We believe it is the first such analysis involving a member of the armoured scale insect family (Diaspididae).

## Methods

### Plant material and insects

Eighteen 2-year-old clonal *A*. *chinensis* ‘Hort16A’ scions were grafted onto 2-year-old clonal *A*. *polygama ‘*Kaimai’ rootstocks in December 2005 at the Plant & Food Research Te Puke Research Centre, Te Puke, New Zealand. Vines were uprooted in July 2007, potted in 30-litre planter bags, pruned and held in a shade house (50% shade) under ambient conditions.

*H*. *lataniae* was reared as pure colonies on potato and butternut squash in a controlled-environment room (20–21°C, 60–80% RH) at the Te Puke Research Centre using a technique similar to that described by Rose [16].

### Microarray

#### Microarray experimental treatments

In the last week of October 2008, 9 weeks after bud break, two actively growing canes (minimum 40 cm long) were chosen on each vine. A length of approximately 20cm was selected on each cane and a settlement aid (wool yarn) wrapped loosely around the cane at a spacing of approximately 1cm. The selected area on both canes of 12 of the vines was seeded with 150 *H*. *lataniae* crawlers (<1-day old) between 1.00 and 3.00 pm on 10 November 2008, using a soft paintbrush to transfer crawlers individually from the insect colony onto each cane. The remaining 12 vines, with wool-wrapped canes but no insects, were used as controls (see example photograph in S1 Fig).

Six of the twelve vines from *H*. *lataniae* treatment and six of the control vines were chosen at random and the bark within the area occupied by the settled insects was sampled after 2 days (12 November 2008 at 1.00–3.00 pm) and 7 days (17 November 2008 at 1.00–3.00 pm). The wool yarn crawler settlement aids were removed and the number of settled first instar insects (white caps) was quickly counted. The wool was left on until the bark was sampled because its removal can cause damage to the delicate scales covering the insects. An approximate 7 cm length of bark was removed from each cane (two per vine) within the region settled by the insects, by making an incision along the length of the cane down to the cambium layer using a scalpel and peeling the bark from the cane with fingers. The bark with its associated scale insects was placed immediately into a labelled plastic vial in liquid nitrogen. Bark removal was completed within 30 seconds from bark incision to immersion in nitrogen, for each sample. The samples were stored at -80°C until extraction. Atmospheric conditions in the shade house, measured by data loggers, varied from 21–31°C and 40–75% RH during the experiment.

#### RNA extraction

Total RNA (mean 26±2.5 (SE) μg/ml) was extracted from 1.5 to 2.5 g of frozen kiwifruit bark using the method of Chang et al. [17]. RNA samples were quantified, and sample purity was verified by using a Nanodrop ND-1000 spectrophotometer (Thermo Fisher Scientific), where 260:280 ratios of 1.8–2.0 were considered of acceptable purity. RNA integrity was checked by an Agilent 2100 analyzer (Agilent Technologies), where non-degraded RNA gave sharp 18S and 28S bands (data not shown).

#### Microarray analysis

17,512 oligonucleotide microarray probes (45–55 bases long, with a consistent melting temperature) were designed using sequence data from the PFR *Actinidia* expressed sequence tag (EST) library [18]. At the time of synthesis of the microarray chip containing all 17,512 probes, this EST database comprised 41,858 non-redundant clusters consisting of 132,577 ESTs. The experimental design for the microarray was a direct comparison of kiwifruit bark tissue inoculated with *H*. *lataniae* or an untreated bark control, 2 and 7 days after inoculation. For each comparison, there were six biological replicates, each duplicated twice in the microarray. cDNA synthesis, labelling, and hybridisation were carried out according to Janssen et al. [19]. Microarray images were converted to 16-bit intensities using GenePix 4000B scanner and Genepix Pro 4.0 software (Molecular Devices). Genepix intensity files were preprocessed and analysed using the limma [20] analysis package in Bioconductor [21]. Data was normalised using the print tip loess method [22], and candidate differentially expressed genes (DEGs) identified using a false discovery rate [23] control set at P < 0.05.

### Gene mining by Basic Local Alignment Search Tool (BLAST), functional annotation and metabolic pathway analysis

Differentially expressed transcripts (P<0.05) were blasted (blastx) against the complete *A*. *thaliana* (TAIR) and *A*. *chinensis ‘*Hongyang’ reference genomes [11,24] (http://bioinfo.bti.cornell.edu/cgi-bin/kiwi/home.cgi) using an expect score cutoff of ≤10^10^.The full list of corresponding orthologues from these two databases, along with Genebank accession numbers is presented in S1 Table. The raw data and experimental details are deposited in GEO. Functional annotation and metabolic pathway analysis was performed using MapMan [25,26], a scavenger gene ontology designed specifically for plant-specific pathways, and an image annotator [26,27]. The scavenger module assigns genes into non redundant and hierarchical (4 tiered) bins. There are 34 top level metabolic pathway bins and approximately 1200 bins at the lowest tiers. The kiwifruit microarray had previously been shown to have a complete and balanced representation of transcripts compared with *A*. *thaliana* and potato [18]. MapMan was used to perform an analysis of changes in expression of putative genes in a bin (pathway) using a Wilcoxon rank sum test with a Benjamini and Hochberg [23] false discovery rate correction that the expression profile of the transcripts in any particular bin was different compared with the remainder of the transcripts on the array. The MapMan image annotator was used to provide visualisation of biotic and abiotic stress-related responses, photosynthesis, secondary metabolism and signalling. To expand understanding beyond the level exposed by the use of this standard annotation tool, we also searched manually and assigned transcripts to pathways and functions based on detailed, literature-based manual annotation and GO ontologies from the *A*. *thaliana* blastx results (http://www.geneontology.org).

### Quantitative PCR (qPCR)

To confirm the gene expression data obtained from the microarray, six genes that were differentially expressed in the microarray experiment (Table 1) were evaluated using qPCR. These genes were selected on the basis of providing a range of up- and down-regulated values in the microarray to determine if the same treatment rankings would occur when using qPCR. Total RNA extracted from kiwifruit bark was treated with deoxyribonuclease I (DNase), Amplification Grade kit (Invitrogen Catalogue No. 18068–015), according to manufacturer’s instructions to remove any genomic DNA. The DNase-treated RNA samples were checked by PCR to confirm that there was no genomic contamination. First-strand cDNA was then synthesised in a 20 μl reaction volume containing 2 μg of DNase-treated RNA, using the Super-Script III first-strand synthesis system for RT-PCR (Invitrogen Catalogue No. 18085–051). Non-template controls included in each PCR plate indicated the purity of the reagents.

**Table 1:** Accession numbers, and primer sequences of target genes (TG) and reference genes (RG) used in real time PCR. Optimal annealing temperatures (T_A_) were predetermined by gradient PCR, and a T_A_ of 55°C was used for all genes.

Gene Name	Genbank accession number	Forward primer (5’-3’)	Reverse primer (5’-3’)
**Endochitinase (TG)**	FG512537	GGTTGCTGCTTTTCTTGCTC	CACAAGGCCACTGTTGATTG
**β-1,3-glucosidase (TG)**	FG455092	TTGGTTCAACATGTCAAAGGAG	TAGGCTGCTTGTTGGGAAAG
**Acyl lipid metabolism (TG)**	FG475093	CAGGATTTAGTAGCAATGATGGAC	AAGGGATCCTCTCGTAATCCA
**TIR-NBS-LRR resistance protein (TG)**	FG525643	GTCTGGCAGGGTTGGTCTTG	GTAACACTGAGGACCGTGCG
**PAD4 triacylglycerol lipase (TG)**	FG479414	ATGCTCGTGACAGGAAACGC	GCAATGCCAATGTAACACCTGC
**CC-NBS-LRR resistance protein (TG)**	FG407814	AGACTTTGAAGATGCCCCCTTGC	TGTAGCCTGCCAATTGACTTTGG
**Actin (RG)**	FG520231	TGCATGAGCGATCAAGTTTCAAG	TGTCCCATGTCTGGTTGATGACT
**Elongation factor EC 3.6.5.3 (RG)**	FG526520	ACAAGCTGGTGACAATGTGG	CGACCACCTTCATCCTTTGT

qPCR was performed in triplicate on RNA from three biological replicates (vines) in 10 μl reactions containing 1 μl of the cDNA (diluted 10-fold in water), 1 μM of each of forward and reverse primers (Table 1) and 5 μl of Light Cycler^®^ 480 SYBR Green 1 Master Mix (Roche Diagnostics GmbH, Mannheim, Germany, Product No. 04 887 352 001). The primers were designed using Primer3 software (The Whitehead Institute, Cambridge, MA, USA) [28] and were synthesised by Invitrogen (Auckland, New Zealand). qPCRs were carried out in a Corbett Rotor-Gene™ 6000 system (Corbett Life Science, Concorde, NSW, Australia). The relative quantification thermal cycling conditions were: denaturation at 95°C for 10 min; followed by 45 cycles of 10 s denaturation at 95°C, 5 s annealing at 55°C and 20 s extension at 72°C. Melting curve analysis (60–95°C at 1°C increments with 5 s between each step) was performed after the final qPCR cycle to validate amplicon specificity. Two reference genes (RGs) that were stably expressed under the conditions of the experiment, actin and elongation factor (EF) were used for normalisation. A gene expression normalisation factor (N), calculated using geNorm software v3.5 [29] for each sample based on the geometric mean of actin and EF was used for the calculation of relative expression of each target gene (TG). Expression of the *H*. *lataniae* treatment is expressed relative to the challenged control RNA samples, which were assigned a value of 1.

### Experiments on the effect of elicitor sprays and a biotrophic pathogen on scale insect growth

*A*. *deliciosa* ‘Hayward’ *v*ines (susceptible to *H*. *lataniae)* at the Plant and Food Research experimental orchard at Ruakura (Hamilton) were sprayed with the salicylic acid mimic Acibenzolar-S-methyl (ASM) at the recommended field application rate equivalent to 1.5 litres per vine (0.2g product per litre) using a hand-held 6 litre garden sprayer on 24/10/2013, 30/10/2013 and 6/11/2013. Samples of canes were taken from six vines on 13/11/2013. Canes were taken into the laboratory and cut into 30 cm lengths which were set upright in individual vials with water and held at 20°C ± 3°C and 50–70% R.H.. Side shoots were removed and wounds were sealed with grafting wax. *H*. *lataniae* crawlers were brushed onto the canes from a laboratory culture grown on squash and allowed to settle under wool yarn wrapped around the cane about 8 times. Details of this method which was developed for germplasm screening for resistance, are published elsewhere [5]. Photographs of the developing scale insects were taken at 1–2 week intervals from 9 January, when the insects were 2 months old, to 21 February 2014 and the areas of the scale insect covers were measured from the photographs using ImageJ (v1.47v) using a method published elsewhere[30].

In a second experiment, the stems of 6-month-old *A*. *deliciosa* ‘Hayward’ seedlings grown from tissue culture were infested with *H*. *lataniae* crawlers (8 plants per treatment). The seedlings were approximately 30cm tall and had 5–8 fully expanded leaves. Two treatments were applied. In the first treatment, Acibenzolar-s-methyl solution (0.1g per litre) was applied to runoff with a hand sprayer on 28 April, 21 May and 11 June 2014. In the second treatment, an aqueous suspension of Psa biovar 3 (10^8^ cfu per ml) was applied to runoff with the same sprayer on 28 April 2014. Plants were inoculated with *H*. *lataniae* on 29 April and held under the same environmental conditions as the first experiment. The size (area) of the *H*.*lataniae* scale cover was assessed using ImageJ software from photographs taken on 9 July 2014 when the insects were 10 weeks old.

## Results

### Scale insect settlement on canes

**Fig 2:**
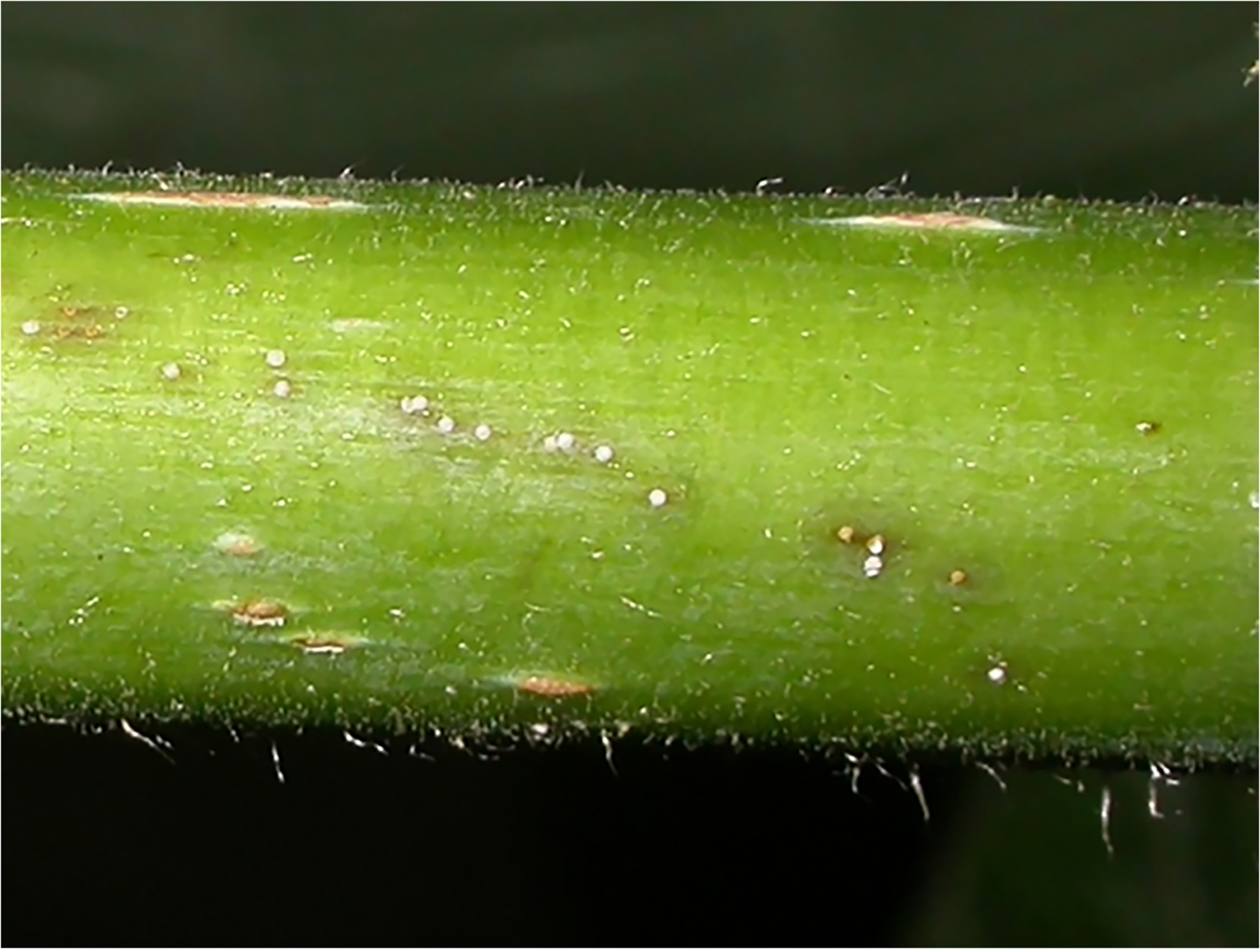
Kiwifruit (*Actinidia chinensis* var ‘Hort16A’) cane (diameter 8.8mm) showing one-week-old 1^st^ instar *H*. *lataniae* scale (‘white caps’–diameter of scale approx. 0.3mm). Note darkening of the tissue surrounding the insects suggesting phenolic/lignin deposition, hypersensitivity (programmed cell death) or both.

The mean number of first instar scale insects settled on the 20 cm length of cane after 2 days was 30 (range 6–70). After 7 days, the mean number of settled first instar scale insects was 65 (range 40–90). *H*. *latania* crawler settlement takes place within 1 day [31] and the numbers counted as settled after 2 days are presumed underestimates due to the accidental removal of the insect cap along with the wool. This effect is less prevalent after 7 days as the caps are larger and better adhered to the cane surface. Thus, the numbers counted on the bark after 7 days are more likely to represent the actual numbers settled in both time treatments. After 7 days, the bark tissue around each scale insect in the *H*. *lataniae* treatment had darkened in response to the presence of the insects in what is assumed to be either a hypersensitive response (programmed cell death) or deposition of phenolics and tannins or both (Fig 2; see [14] and S2 Fig for more detailed pictures from a previous experiment). The tissue darkening was also visible on the inside of the bark after its removal from the cane.

### qPCR versus microarray results

**Fig 3:**
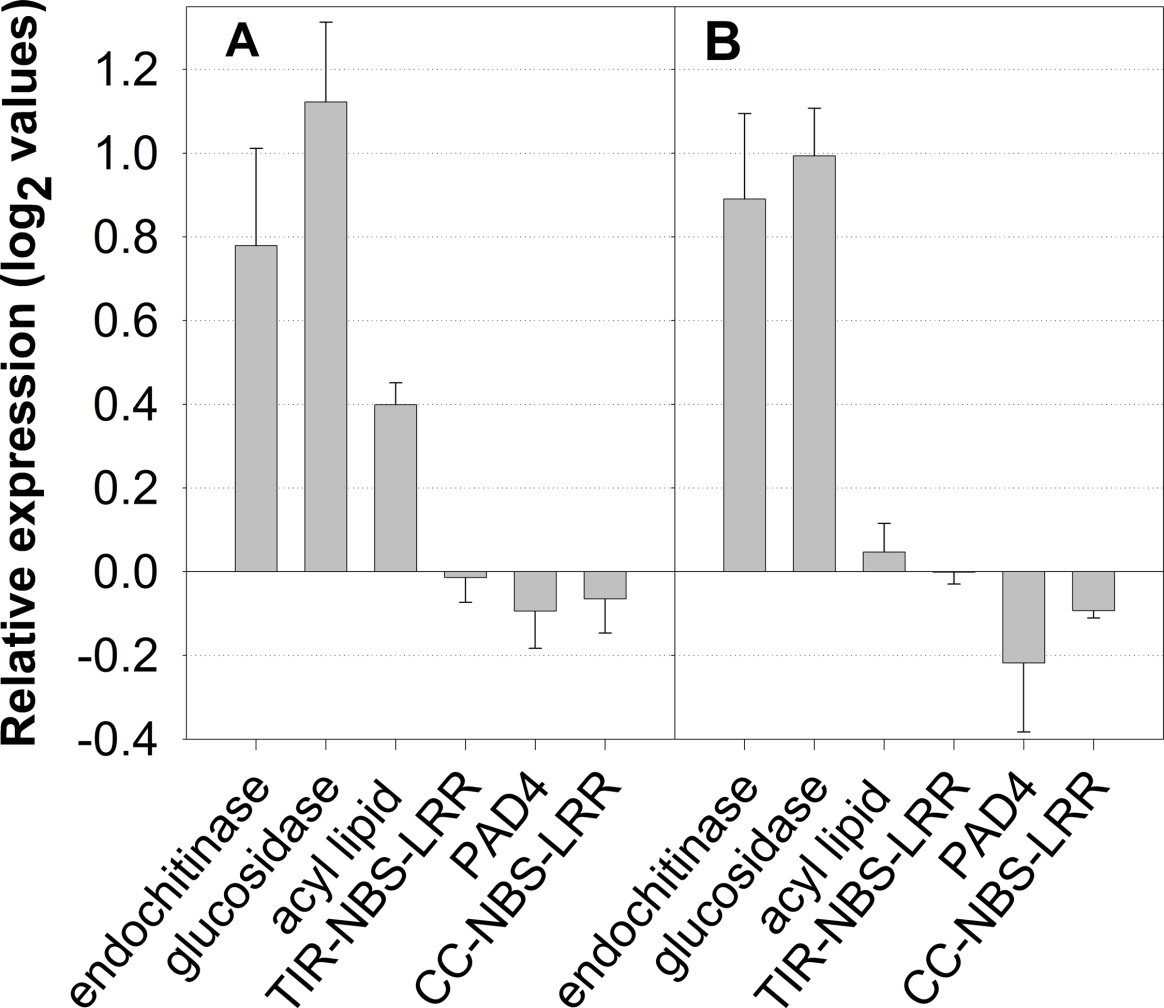
Comparison of quantitative polymerase chain reaction (qPCR) and microarray results for relative expression of six differentially expressed genes: endochitinase; β-1,3-glucosidase (glucosidase), a protein involved in acyl lipid metabolism (acyl lipid); a leucine rich repeat (LRR) putative resistance (R) gene with a TIR domain (TIR-NBS-LRR), phytoalexin deficient 4 (PAD4), and an LRR putative R gene with a CC domain (CC-NBS-LRR). **A)** 7-day *H*. *lataniae—*control treatment comparison, qPCR data; **B)** 7-day *H*. *lataniae—*control comparison, microarray data. For qPCR, all data are normalised against expression of actin and elongation factor. Error bars present standard errors of the mean.

There was close agreement between the qPCR and microarray expression data, with both methods of expression analysis ranking the genes in the same relative order in terms of up- and down-regulation (Fig 3).

### Microarray analysis of differential gene expression in response to *H*. *lataniae* feeding

**Fig 4:**
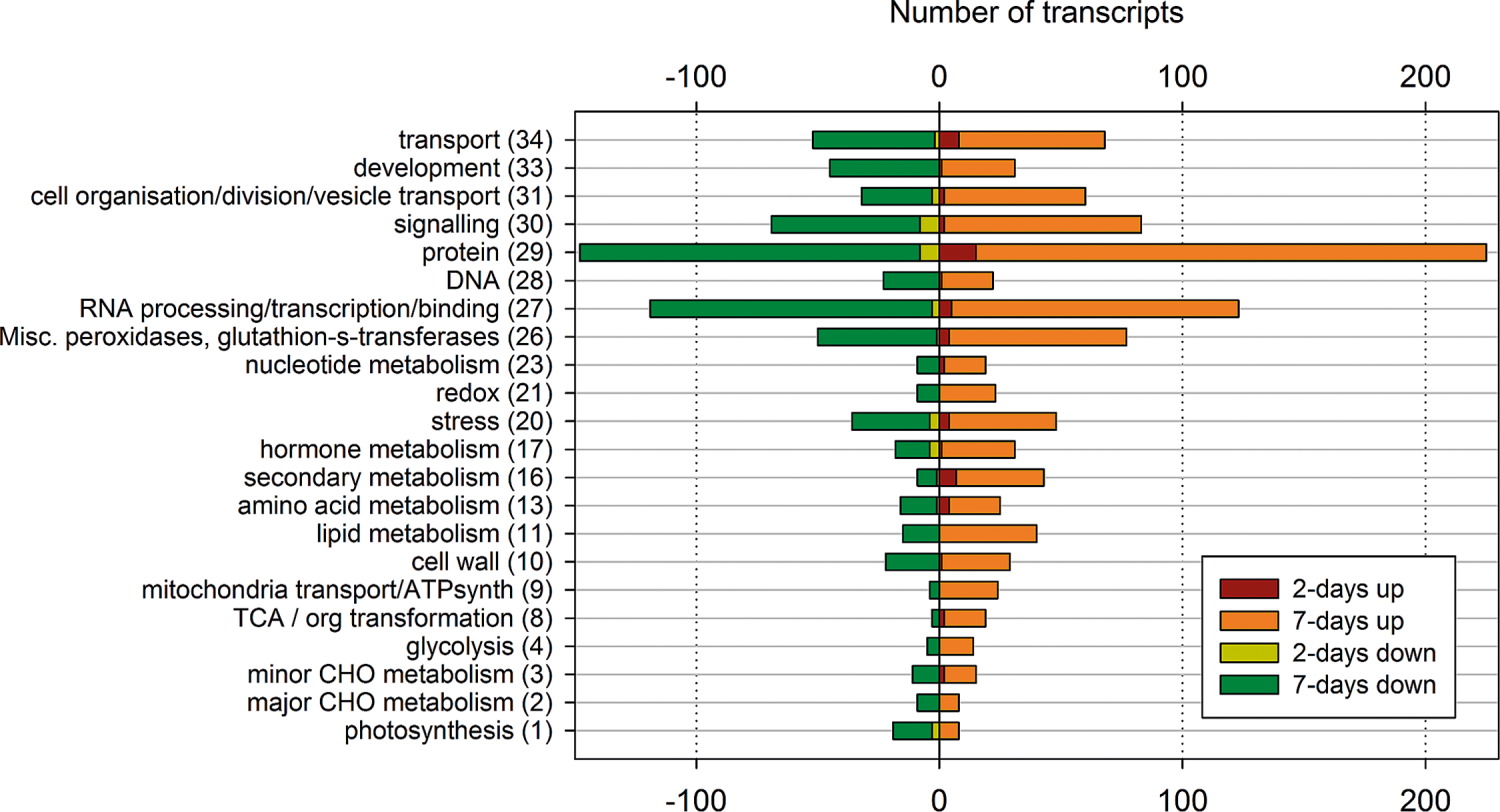
Numbers of differentially expressed transcripts categorised into major MapMan bin groups (bin numbers in brackets) from *A*. *chinensis* ‘Hort16A’ canes after 2-days and 7-days exposure to *H*. *lataniae*. Down-regulated transcrips are negative. Unassigned transcripts were 321 and -298 after 7 days and 24 and -9 after 2 days.

Analysis of the microarray data from kiwifruit bark showed that 2 days after transfer of scale insects to the canes, 272 genes were differentially expressed (of which 66% were up-regulated), 51 to a log_2_ fold change ≤-0.585 or ≥0.585 (76% up-regulated). After 7 days, 5,284 (30%) of 17,512 gene transcripts were differentially expressed, (55% were up-regulated), 850 (77% up-regulated) to a log_2_ fold change ≤-0.585 or ≥0.585. MapMan mapped 2,458 of the differentially expressed ESTs after 7 days and 139 of 272 differentially expressed ESTs after 2 days into over 1,000 bins. All of the 34 major bins were represented and most of the sub-bins (Fig 4).

**Fig 5:**
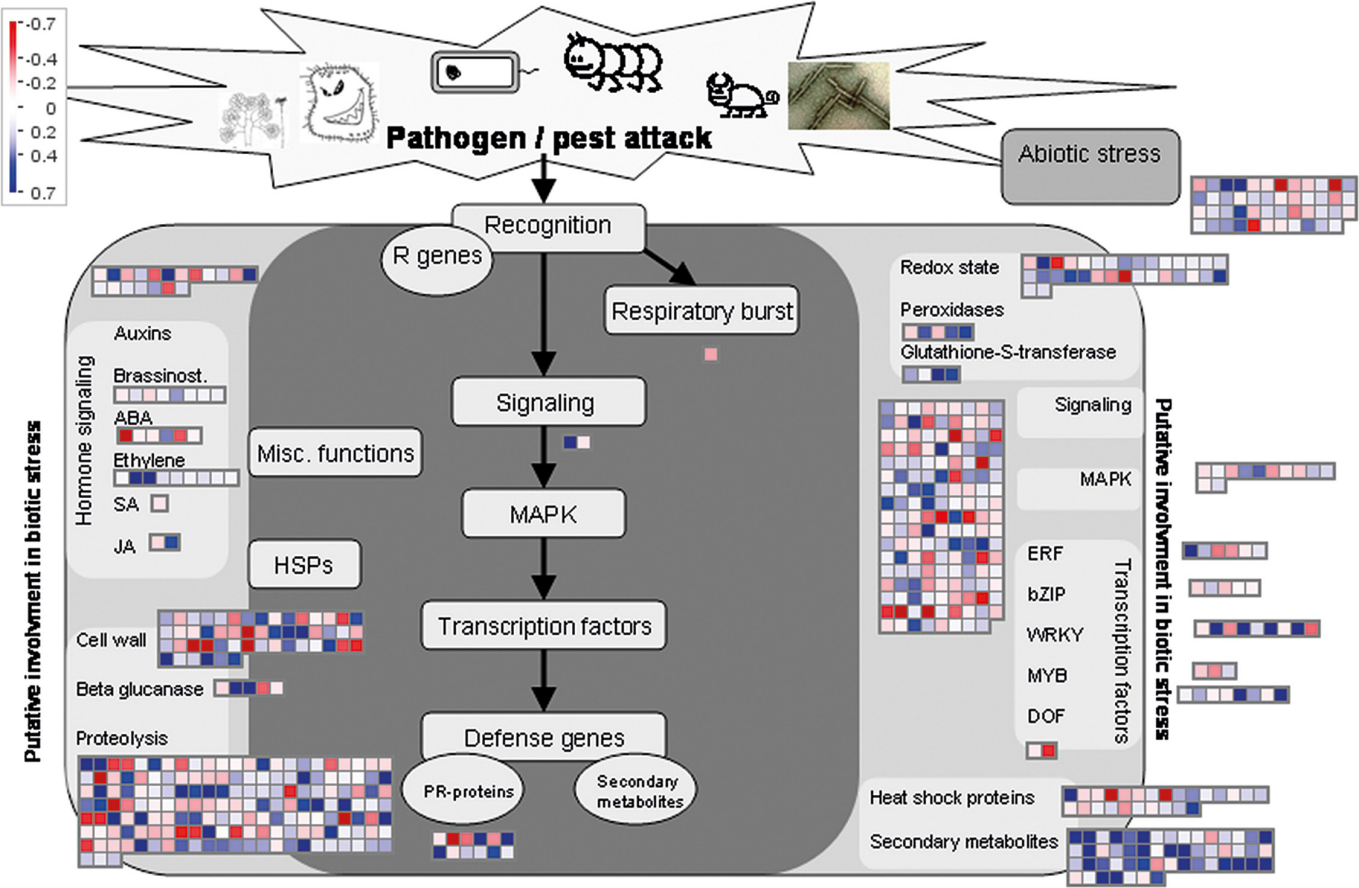
MapMan analysis of changes in biotic stress-associated transcript expression after 7 days in response to *H*. *lataniae* feeding. Transcripts in the dark grey panel are orthologous with genes having experimental indication of involvement with biotic stress responses [27]. Transcripts in the light grey panels on either side are orthologous with genes that are putatively associated with biotic stress. Coloured squares represent transcripts differentially expressed in response to *H*. *lataniae* feeding on kiwifruit cane bark expressed as the ratio of insect-challenged cane bark tissue compared with bark from control canes. The colour scale is shown in top left: red down-regulated; blue, up-regulated on a log_2_ scale. Full list of transcript descriptions is given in S1 Table and summary in Table 2.

MapMan identified 604 differentially expressed transcripts putatively associated directly or indirectly with biotic stress (Fig 5; S1 Table). A further 39 transcripts were identified from manual inspection and blastx against the TAIR database (S1 Table). A summary of the functional annotation of these 643 transcripts (Table 2) shows 49 were directly related to biotic stress (BIN 20.1) and 51 to abiotic stress (BIN 20.2). 21 of the biotic stress-related transcripts code for pathogenesis related proteins (BIN 20.1.7), six of which are putative PR-1 and PR-2 (e.g. *BGL2* β 1,3 glucosidase) proteins (Table 2; S1 Table). Eight transcripts mapping to NBS-LRR signalling (R) genes were differentially expressed (S1 Table). One transcript mapping to lipoxygenase (LOX2) (BIN 17.7.1.2), a marker for jasmonic acid synthesis [32], was down-regulated.

**Table 2:** Summary of numbers of differentially expressed stress-related transcrips from kiwifruit (*Actinidia chinensis* ‘Hort16A’) grouped by the first two layers of MAPMAN bin codes (first 3-layers for bin 20 stress-related) after 2- and 7-days exposure to feeding by *H*. *lataniae*. **Details of the transcripts and orthologues are given in** S1 **Table.**

		7-days		2-days	
BIN	BIN label	up	down	up	down
10.1	cell wall.precursor synthesis	7	4		
10.2	cell wall.cellulose synthesis	2	3		
10.3	cell wall.hemicellulose synthesis	1	5	1	0
10.5	cell wall.cell wall proteins	5	2		
10.6	cell wall.degradation	4	5		
10.7	cell wall.modification	4	3		
10.8	cell wall.pectin*esterases	6	0		
16.1	secondary metabolism.simple phenols	15	1	3	0
16.2	secondary metabolism.phenylpropanoids	12	3	0	1
16.4	secondary metabolism.N misc	1	1		
16.5	secondary metabolism.sulfur-containing	4	0	1	0
16.7	secondary metabolism.wax	1	0		
16.8	secondary metabolism.flavonoids	9	2	1	0
16.99	secondary metabolism.unspecified	1	1		
17.1	hormone metabolism.abscisic acid	1	5		
17.2	hormone metabolism.auxin	11	8	0	1
17.3	hormone metabolism.brassinosteroid	6	2		
17.5	hormone metabolism.ethylene	10	0	1	1
17.7	hormone metabolism.jasmonate	3	1		
17.8	hormone metabolism.salicylic acid	1	1		
20.	stress	0	2		
20.1	stress.biotic	10	5	2	0
20.1.1	stress.biotic.respiratory burst	1	1		
20.1.3	stress.biotic.signalling	4	3	1	1
20.1.4	stress.biotic.kinases	4	0	2	0
20.1.7	stress.biotic.PR-proteins	14	5	5	0
20.2	stress.abiotic.	3	1	1	1
20.2.1	stress.abiotic.heat	14	11	1	0
20.2.2	stress.abiotic.cold	3	1		
20.2.3	stress.abiotic.drought/salt	7	3	0	1
20.2.4	stress.abiotic.touch/wounding	1	1		
20.2.5	stress.abiotic.light	0	1		
20.2.99	stress.abiotic.unspecified	4	1	0	1
21.1	redox.thioredoxin	7	4		
21.2	redox.ascorbate and glutathione	9	1		
21.3	redox.heme	0	1		
21.4	redox.glutaredoxins	1	1		
21.5	redox.peroxiredoxin	1	1		
21.6	redox.dismutases and catalases	2	1		
21.99	redox.misc	3	0		
26.12	misc.peroxidases	4	2		
26.4	'misc.beta 1,3 glucan hydrolases'	0	3		
26.9	misc.glutathione S transferases	5	0		
27.3	'RNA.regulation of transcription	15	18		
29.5	protein.degradation	91	72	4	1
30.1	signalling.in sugar and nutrient physiology	2	6		
30.11	signalling.light	8	10	0	4
30.2	signalling.receptor kinases	22	21	0	1
30.2.11	Class XI (incl. PRRs PEPR1/2)	1	3		
30.2.12	Class XII (incl. PRRs FLS2, EFR)	0	1		
30.3	signalling.calcium	18	6	1	1
30.4	signalling.phosphinositides	3	9	0	1
30.5	signalling.G-proteins	18	10	1	0
30.6	signalling.MAP kinases	6	6		
30.7	signalling.14-3-3 proteins	2	0		
30.8	signalling.misc	1	0		
30.9	signalling.lipids	0	1		
30.99	signalling.unspecified	1	0		

Secondary metabolism (BIN 16) was showing signs of up-regulation after 2 days and was strongly up-regulated after 7 days (Tables 2 & 3; S3 Fig; S1 Table). Transcripts associated with the phenylpropanoid pathway and lignin synthesis (S1 Table; S4 Fig) were significantly up-regulated. Transcripts of enzyme orthologues significantly up-regulated include Phenylalanine ammonia lyase (FG 484623, FG511928), Chalcone synthase (FG528287), Coumarate co-A ligase (FG436255, FG515208), Caffeoyl-CoA 3-O-methyltransferase (FG528071) and Cinnamoyl-CoA reductase (FG490671, FG397115) (Table 2; S4 Fig). After 2 days, 3 transcripts from the mevalonate pathway were strongly up- regulated (FG521975, FG18347, FG471315). They were also strongly up-regulated after 7 days (S3 Fig; S1 Table).

**Table 3:** Significantly altered processes of protein families according to changes in gene expression level in *A*. *chinensis* canes challenged by *H*. *lataniae* after 2- and 7-days. Numbers shown are genes annotated to each process or family according to MAPMAN ontology. P-values are from a Wilcoxon rank sum test.

MapMan BIN	Name	Up regulated	Down regulated	p-value
**2-days**				
30.11	Signalling.light	0	4	0.16
**7-days**				
1	Photosynthesis	9	19	0.0990
1.1	Photosynthesis. lightreaction	4	14	0.0427
1.1.1	Photosynthesis. lightreaction.photosystem II	0	10	0.0005
1.1.1.2	Photosynthesis. Lightreaction.ps II polypeptide subunits	0	6	0.0538
16	Secondary metabolism	43	9	0.0004

Light signalling was showing signs of down-regulation after 2 days (Table 3), and photosystem II genes were significantly down-regulated after 7 days (Table 3, S1 Table; S5 Fig). 150 differentially expressed transcripts associated with stress-related signalling were identified (S1 Table; S6 Fig). This included a group of 43 putative defence-related signalling receptor kinase transcripts (S6 Fig). Five of these belong to BINs 30.2.11 and 30.2.12 known to contain recognised PRRs (Table 3), while a further three RLK-LRR transcripts (FG528482, FG525734, FG423513) from BIN 20 are orthologous with defence-related genes (Tables 2; and S1). Transcript FG496474 is orthologous with the hub protein encoding gene *AtTCP14* (At3G47620) that has been reported to act as a central hub for convergent pest/pathogen effectors and plant immune responses [33].

### Experiments on the effect of elicitor sprays and a biotrophic pathogen on scale insect growth

**Fig 6:**
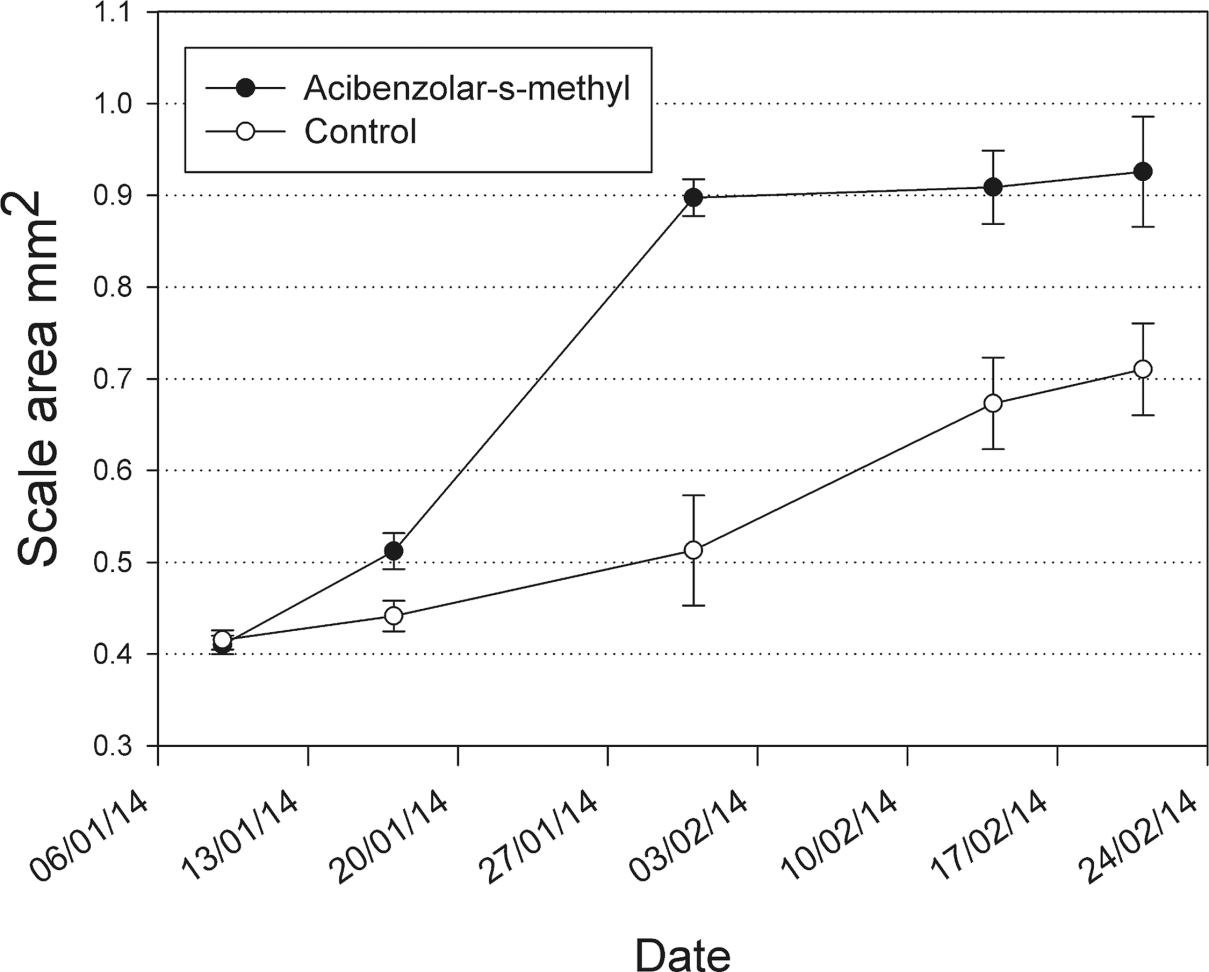
Mean (±SE) scale cap area for *H*. *lataniae* growing on untreated *A*. *deliciosa* canes (solid circles) and acibenzolar-s-methyl treated canes (open circles).

In the cut cane bioassays, *H*. *lataniae* grew more slowly and was significantly smaller after 9 weeks on the acibenzolar-s-methyl treated canes than scale on canes from untreated vines (Fig 6). The final average size of the *H*. *lataniae* scales from the acibenzolar-S-methyl treated canes after 14 weeks (0.71 mm^2^) was 23% smaller in area than scales from the control vines (0.93mm^2^).

In the second laboratory experiment, after 10 weeks, *H*. *lataniae* growing on the stems of acibenzolar-s-methyl treated whole plants were 16% smaller (one-way ANOVA and Dunnett’s test with 5% family error rate) than those on the control plants; however scale insects growing on petioles and leaf nodes were the same size in treatments and control (Table 4).

**Table 4:** The mean area (mm^2^) of 10-week old *H*. *lataniae* adult armoured caps from insects settling on the stems, petioles and leaf nodes of potted *A*. *deliciosa* ‘Hayward’ plants subjected to three Acibenzolar-s-methyl (ASM) sprays and *Psuedomonas syringae* pathovar actinidiae (Psa) inoculation compared with those on untreated control plants. Sample sizes in brackets. The mean size of the insects on ASM-treated plants was significantly smaller than the insects on control plants (Dunnett’s test with family error rate of 5%), but insects on Psa-treated plants were not different from those on stems of control plants.

	Mean (n) *H*. *lataniae* size (mm^2^) on organ type		
**Treatment**	stem	petiole	node
**Control**	1.28 (42)	1.80 (17)	1.27 (11)
**Psa**	1.10 (42)	1.88 (18)	1.36 (16)
**ASM**	1.08 (37)	1.77 (37)	1.26 (7)
**Pooled SD**	0.38	0.437	0.31
**P (one-way ANOVA on column means)**	0.036	0.71	0.69

## Discussion

While acknowledging that gene expression does not necessarily correspond to changes in protein levels, the results of the microarray experiment provide preliminary evidence for the reprogramming of *A*. *chinensis* gene expression in response to first instar *H*. *lataniae* feeding, suggesting an increase in stress responses and secondary metabolites concomitant with a decrease in photosynthesis. Although scale-feeding does not cause extensive physical damage to the plant, statistically significant reprogramming of 30% of transcript expression had occurred 7 days after *H*. *lataniae* feeding. The level of fold changes was modest (generally log_2_≤1.6), but this was most likely due to the relatively small amount of cane tissue challenged by the armoured scale insect feeding (average of 65 insects settled per cane—~one per 30 mm^2^) and the very small size of the neonate *H*. *lataniae* (0.3–0.5mm long).

MapMan analysis of pathway changes showed that photosynthesis (photosystem II; BIN1) was down-regulated and secondary metabolism (BIN 16) was up-regulated after 7 days. In addition, there were substantial changes to expressions of stress-related transcripts associated with cell wall metabolism (BIN 10), hormone metabolism (BIN 17), biotic and abiotic stress (BIN 20), redox reactions (BIN 21), peroxidase metabolism (BIN 26) and signalling (BIN 30, notably BIN 30.2 –receptor kinases (RLK-LRR)). Photosynthesis has been found to be down-regulated in response to water and drought stress, pathogen and insect attack, exogenous application of salicylic acid and decreases in most incompatible plant-pathogen interactions [34,35]. Secondary metabolism plays an important role in plant defence against insect herbivory, with many secondary metabolites functioning in both direct defence as toxins, repellants and anti-digestive compounds, and in indirect defence as volatile compounds that attract predators[36]. The up-regulation of transcripts associated with the phenylpropanoid pathway (e.g. PAL, Chalcone synthase (CHS), Cinnamoyl Co-A reductase (CCR1); 4Coumarate CoA-ligase (4CL1); Elicitor-activated gene (ELI3); BIN 16.2), suggests that after 7 days the cane was mounting a robust defence against *H*. *lataniae* feeding. As well as being responsible for production of many secondary metabolites, PAL and chalcone synthase are also involved in the synthesis of SA [37,38]. Up-regulation of PAL has also been associated with salicylic-induced induction of resistance in ‘Hort16A’ kiwifruit to bacterial canker [39,40].

99 transcripts associated directly with biotic or abiotic stress (BIN 20) were differentially expressed. Several of these (e.g. PR1, BGL2) are known to function as important markers of SA-induced resistance [41–43]. One of the most strongly up-regulated PR2 transcripts (FG455092) has been shown to be a reliable marker associated with SA-induced resistance to ripe rot caused by the fungal pathogen *Cryptosporiopsis actinidiae* on *A*. *chinensis* ‘Hort16A’ [44,45] and against the bacterial pathogen *Pseudomonas syringae* pv. *actinidiae* (Psa) on the same kiwifruit cultivar in a range of qPCR and next generation sequencing experiments (Wurms, unpublished data). It has been hypothesized that this enzyme facilitates a quick plant response to biotic invasion by converting preformed inert phytoanticipins (synthesized via the phenylpropanoid pathway) into their corresponding toxic aglycones [46]. Another up-regulated PR2 transcript (FG480859) is orthologous with AT3G57260 (S1 Table), coding for a protein that has been used as a marker of SA-dependent responses of *Arabidopsis* to aphid feeding [42].

Several transcripts orthologous with genes coding for JA-mediated defence were differentially expressed, most being down-regulated. The antagonistic effect of SA on the JA pathway is often expressed at the level of gene transcription and JA transcription may be targeted by SA [32,47]. For example, four up-regulated transcripts (FG514473, FG500231, FG468929, FG474406) are orthologous with WRKY transcription factor reported as playing a role in SA-mediated suppression of JA-responsive genes [32]. Up-regulation of FG460267, an orthologue of the redox regulator At Glutathion S-transferase is also associated with increase in SA signalling and reduction in JA in *A*. *thaliana* [48]. A transcript encoding LIPOXYGENASE 2 (orthologue of the Hongyang protein Achn123621), the principal lipoxygenase mediator of *A*. *thaliana* JA biosynthesis [43], was also down-regulated in the current study.

Eight NBS-LRR (putative resistance gene) orthologous transcripts were differentially expressed along with transcripts putatively associated with programmed cell death (FG398641) and the regulation of hypersensitive response and SA-signalling (FG431814) (BIN20). The response of *A*. *chinensis* ‘Hort16A’ to *H*. *lataniae* feeding has been described elsewhere [14] and shows some morphological characteristics consistent with a hypersensitive response (localised cell death, phenolic and lignin/callose deposition in addition to wound periderm formation) [49–51], but over a longer time scale (1–4 weeks) than is typical of HR responses reported against plant pathogens e.g.[50,51]. Further work is required to determine whether these substantial physical changes are directly analogous with a hypersensitive response and triggered by R-gene protein signalling.

The putative reprogramming of primary carbon metabolism between photosynthesis (down-regulation of transcripts associated with photosystem II–BIN 1.1) and cell wall strengthening (BIN 10) observed in this study has been noted frequently in response to fungal, bacterial and virus attack [52–54], and less commonly following insect attack [55]. The concept of resistance induction coming at a physiological cost to plant, is widely accepted, regardless of the type of biotic or abiotic elicitor [56].

Recent research has indicated that independently specialised effector proteins from a wide range of organisms interact with a limited set of highly connected host proteins, which act as cellular hubs of the plant immune system [57]. Diverse effectors interacting with R genes converge on protein hubs which in turn interact with large numbers of host proteins, resulting in a plethora of chemical reactions. The discovery of an orthologue (FG496474) to the AtTCP14 hub protein (a transcription factor which functions via interactions with diverse proteins modulating cell metabolic homeostasis) will assist with ongoing studies into plant immune responses in *A*. *chinensis*. Since hub proteins are thought to interact indirectly with effectors, this suggests possible involvement of an ETI response. Another transcript (FG514166) orthologous with an up-regulated biotic stress protein RIN4, a well-known regulator of plant immunity which is targeted by bacterial effectors and controls aspects of both PTI and ETI responses, is up-regulated in the current study. Current evidence however suggests that post-translational regulation of this gene product may play an important role [58,59] and it remains to be seen how important this transcriptional control is. Confirmation of an ETI response based on transcriptional analysis alone is not possible, and requires proof of specific plant R genes that interact with insect effectors. The differential expression of five transcripts (four down-regulated and one up-regulated) associated with receptor like kinases that are PRR candidates (BINs 30.2.11 and 30.2.12) after 7 days supports the view that PTI may also be important for *A*. *chinensis* defence [11]. However the supposition that all 20 transcripts orthologous with putative RLK-LRR genes in *A*. *chinensis* ‘Hongyang’ are associated with PTI [11] is unlikely and will require further research to uncover their function. In their presentation of the *A*. *chinensis* Hongyang genome, Huang et al. [11] suggested that the large number of identified RLK-LRR genes (261) compared with R-genes (96) was indicative of an enhanced role for PTI in the plant’s defence systems. However as RLK-LRR genes play a role in a diverse range of cellular processes, a more conservative approach would be to associate this putative function only with genes belonging to RLK-LRR branches known to contain functionally characterised PRR genes such as FLS2, EFR and PEPR1/2.

WRKY transcription factors play a key role in the transcription of genes associated with both PTI (‘basal’ immune responses) and ETI responses [32,60,61]. They are involved in both positive and negative SA signalling. Four transcripts orthologous with defence-related WRKY transcription factors were all up-regulated. FG468929 is orthologous with *AtWRKY33* (At2g38470) which has been shown to be up-regulated in response to salt stress, pathogen attack and senescence [60,62], and to have a central signalling function in basal defence-related (PTI) responses to necrotrophic pathogens [63–65]. Up-regulation of *AtWRKY33* and *AtWRKY75* (orthologous with FG474406) has been associated with a PTI-response of *A*. *thaliana* to feeding by the aphid *Brevicoryne brassicae* [66]. *At*WRKY75 and *At*WRKY28 (FG514473 orthologue) are reported to be important for *A*. *thaliana* defensive responses (PTI-response) to necrotrophic pathogens [60,67]. *AtWRKY75* up-regulation has also been associated with hydrogen peroxide-induced cell death [68]. In strawberry, the *AtWRKY75* orthologue has been shown to be associated with both PTI- and ETI-responses, and defence against the aphid *B*. *brassicae* [69].

MAPK signalling cascades are important mediators of hormonal changes in eukaryotes [70], including the mediation of PTI- and ETI-recognition responses in plants [71–74]. Several up-regulated *At*MAPK orthologous transcripts are associated with both PTI- and ETI- defence responses. FG511331 is orthologous with *AtMAPK4*, which is associated with PTI- and ETI-response signalling [70,71,74,75] and has been shown to be associated with long-lasting resistance to pathogen attack in *A*. *thaliana* plants [76]. FG482696, which was up-regulated after 2 and 7 days, is orthologous with *A*. *thaliana phospholipase A IIA* gene (At2G26560) which is associated with multiple responses to biotic and abiotic stress, SA and JA signalling and apoptosis [77]. FG517120 is orthologous with *A*. *thaliana MKS1*, a nuclear substrate of AtMAPK4 which is involved in phytoalexin production [78]. FG460354 is an orthologue of Coronatin Insensitive 1 (*COI1*) and is down-regulated in this study. COI1 is the receptor for JA-Isoleucine, the most active JA metabolite, and as such is a key regulator of the jasmonic acid defence pathway. COI1 is associated with defence against nectrotrophic pathogen attack [32,65], enhanced resistance to biotrophic pathogens and amelioration of MAPK4 and *At*WRKY33 (an orthologue of FG468929) activity in a cascade producing phytoalexins in *A*. *thaliana* [76,78,79].

Supposition from the transcriptional data of an SA-pathway mediated defence response is supported by the outcome of the experiments to investigate the effect of exogenous SA application to *A*. *deliciosa* plants on *H*. *lataniae* growth in which the SA-mimic Acibenzolar-S-methyl reduced the growth of *H*. *lataniae* on *A*. *deliciosa* cut canes and the stems of whole *A*. *deliciosa* plants. The lack of effect of the SA-mimic (and the bacterial infection) on the growth of scale insects growing on the petioles and leaves may reflect differences in gene expression on these plant parts. Previous studies of *H*. *lataniae* growth on resistant *A*. *deliciosa* genotypes have shown complete resistance of the plant and death of *H*. *lataniae* on the stem but normal insect survival and growth on adjacent petioles (Hill, Mauchline unpublished observation; S7 Fig). This differential response by the plant to the insect attack still confers resistance as it is deciduous and sheds the slow growing, sessile insects with its leaves in autumn. Confirmation of this putative SA-mediated kiwifruit defence against *H*. *lataniae* will require measurements of host protein and hormone levels as well as examination of the effect of knock-out mutants. More studies of compatible and incompatible plant responses to diaspid feeding will be required before meaningful comparisons can be drawn between plant defence responses against this important sucking insect family with other plant-sucking pests (e.g. aphids, whitefly, mites).

This paper reports on the first study of transcriptional responses of resistant *A*. *chinensis* ‘Hort16A’ kiwifruit to *H*. *latania* feeding. Results showed a decrease in photosynthesis and an increase in stress responses, possibly involving ETI and PTI, with predominance of the SA signalling pathway. Lab and field trials confirmed that *H*. *latania* growth *in planta* was adversely affected by application of an SA-mimic. This information will be used to design further trials to refine our understanding of resistance to *H*. *latania*, with a view to incorporating resistance to this pest into future kiwifruit varieties.

## Supporting Information

S1 Fig Kiwifruit canes set up for a resistance bioassay showing wool wrapped around the cane as an aid to crawler settlement. The close up shows newly settled, 1-week-old, *Hemiberlesia lataniae* first instar “white caps” that settled beneath the wool, which has been carefully moved aside to reveal the insects. The canes diameters are 12–18mm.
(JPG)

S2 Fig Response of young (4-month-old) *Actinidia chinensis* cane six weeks after settlement of *Hemiberlesia lataniae* showing cell death around the insect (1^st^ instar nymph). Approximate diameter of white cap is 0.5mm. Further analysis of the plant response can be found at Hill et al. 2011 The response of resistant kiwifruit to armoured scale insect feeding, Arthropod Plant Interactions; DOI 10.1007/s11829-011-9124-9
(PNG)

S3 Fig Mapman- based visualisation of the transcripts involved in secondary metabolism (BIN 16) in *Actinidia chinensis “*Hort16A” kiwifruit 7 days after challenge by *H*. *lataniae*. Red squares denote down-regulated transcripts and blue, upregulated. 62 data points (transcripts) mapped. Secondary metabolism is significantly up-regulated. See also Table 2 and S1 Table for details of transcripts.
(PNG)

S4 Fig Mapman- based visualisation of the transcripts involved in the phenylpropanoid pathway (BIN 16.2) in *Actinidia chinensis “*Hort16A” kiwifruit 7 days after challenge by *H*. *lataniae*. 28 data points (transcrips) are mapped and the pathway is significantly up-regulated. Red squares denote down-regulated transcripts and blue, upregulated. See text for details and S1 Table for transcript information.
(PNG)

S5 Fig MapMan visualisation of Photosynthesis pathway (BIN 1) in *Actinidia chinensis “*Hort16A” kiwifruit 7 days after challenge by *H*. *lataniae*. 25 data points mapped. Photosystem II pathway was down-regulated after 7 days. Red squares denote down-regulated transcripts and blue, upregulated. See paper text for details and S1 Table for transcript information.
(PNG)

S6 Fig MapMan visualisation of differentially expressed transcripts of receptor-like kinases (BIN 30) in *Actinidia chinensis “*Hort16A” kiwifruit 7 days after challenge by *H*. *lataniae*. Note activity of RLK-LRR kinases (BIN 30.2) on extreme left. Red squares denote down-regulated transcripts and blue, upregulated. See text for details and S1 Table for transcript information.
(PNG)

S7 Fig 9-week old armoured scale insect, *Hemiberlesia lataniae*, on the stem and leaf petiole of a small potted experimental *Actinidia deliciosa* plant showing differential response of plant parts to insect feeding. The stem is resistant to *H*. *lataniae*, but the petiole is susceptible. Note the small, dead insects on the stem (red arrows) surrounded by dead cells compared with the much larger, live insect still growing on the petiole (blue arrow). The stem is approximately 1.2cm in diameter. This could be an optimal defence strategy for a deciduous plant against a sessile pest. [black marks are marker pen].
(PNG)

S1 Table Table of differentially expressed stress-related transcripts from kiwifruit (*Actinidia chinensis* 'Hort16A') defined by MAPMAN bin codes after 2- and 7-days exposure to feeding by *Hemiberlesia lataniae*. (XLSX)
