# Supplementary figures and images for: Transcriptome Analysis of Kiwifruit (Actinidia chinensis) Bark in Response to Armoured Scale Insect (Hemiberlesia lataniae) Feeding
Source: PLoS One. 2015 Nov 16;10(11):e0141664. doi: 10.1371/journal.pone.0141664 (PMC4646472; doi:10.1371/journal.pone.0141664)

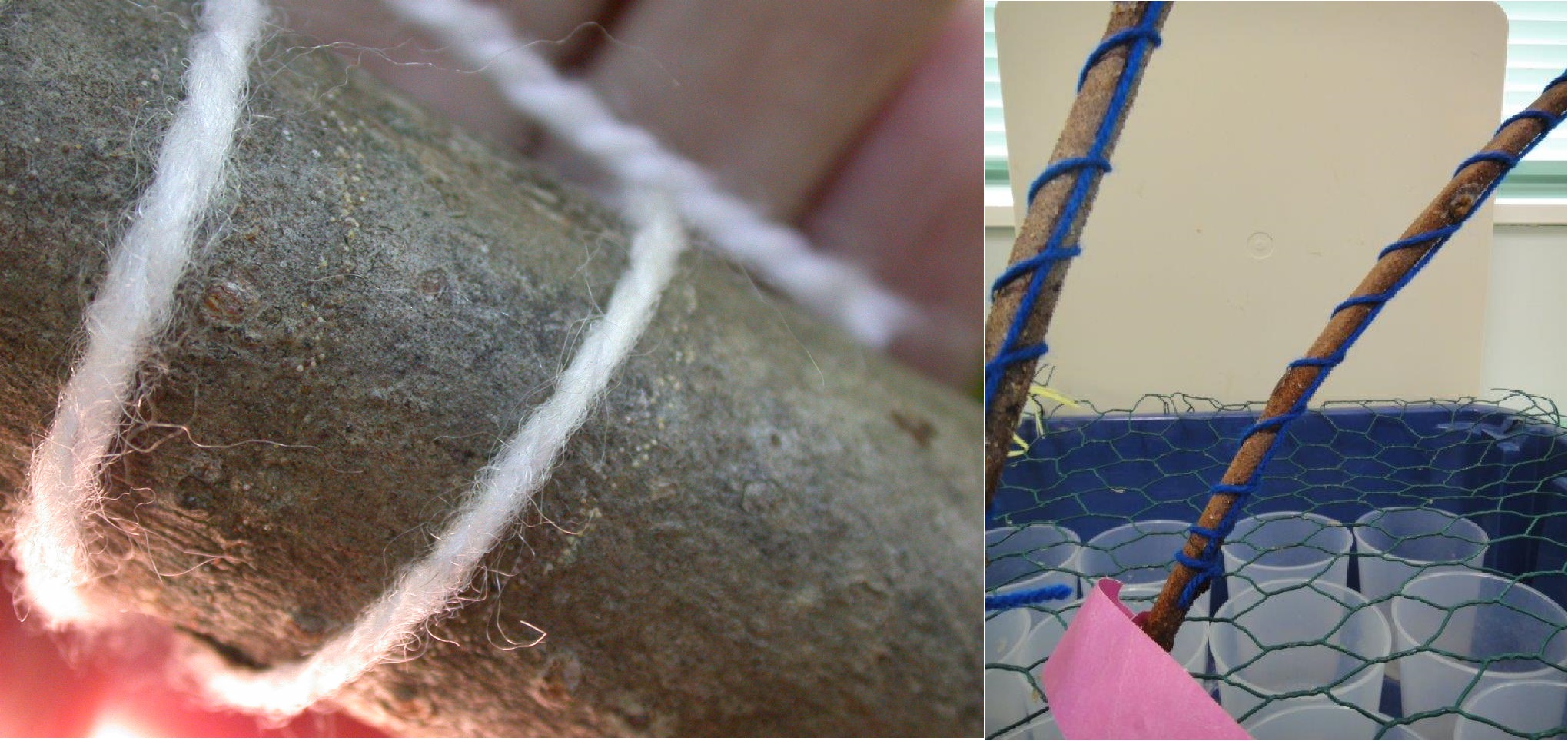

Supplement: S1 Fig — The close up shows newly settled, 1-week-old, Hemiberlesia lataniae first instar “white caps” that settled beneath the wool, which has been carefully moved aside to reveal the insects. The canes diameters are 12–18mm. (JPG) [file pone.0141664.s001.JPG]

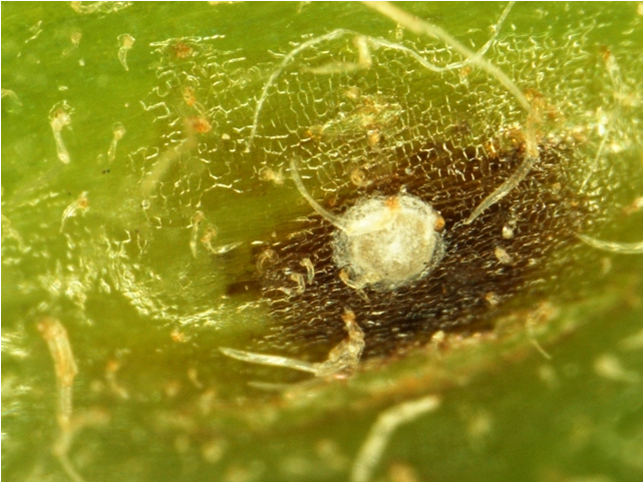

Supplement: S2 Fig — Approximate diameter of white cap is 0.5mm. Further analysis of the plant response can be found at Hill et al. 2011 The response of resistant kiwifruit to armoured scale insect feeding, Arthropod Plant Interactions; DOI 10.1007/s11829-011-9124-9 (PNG) [file pone.0141664.s002.png]

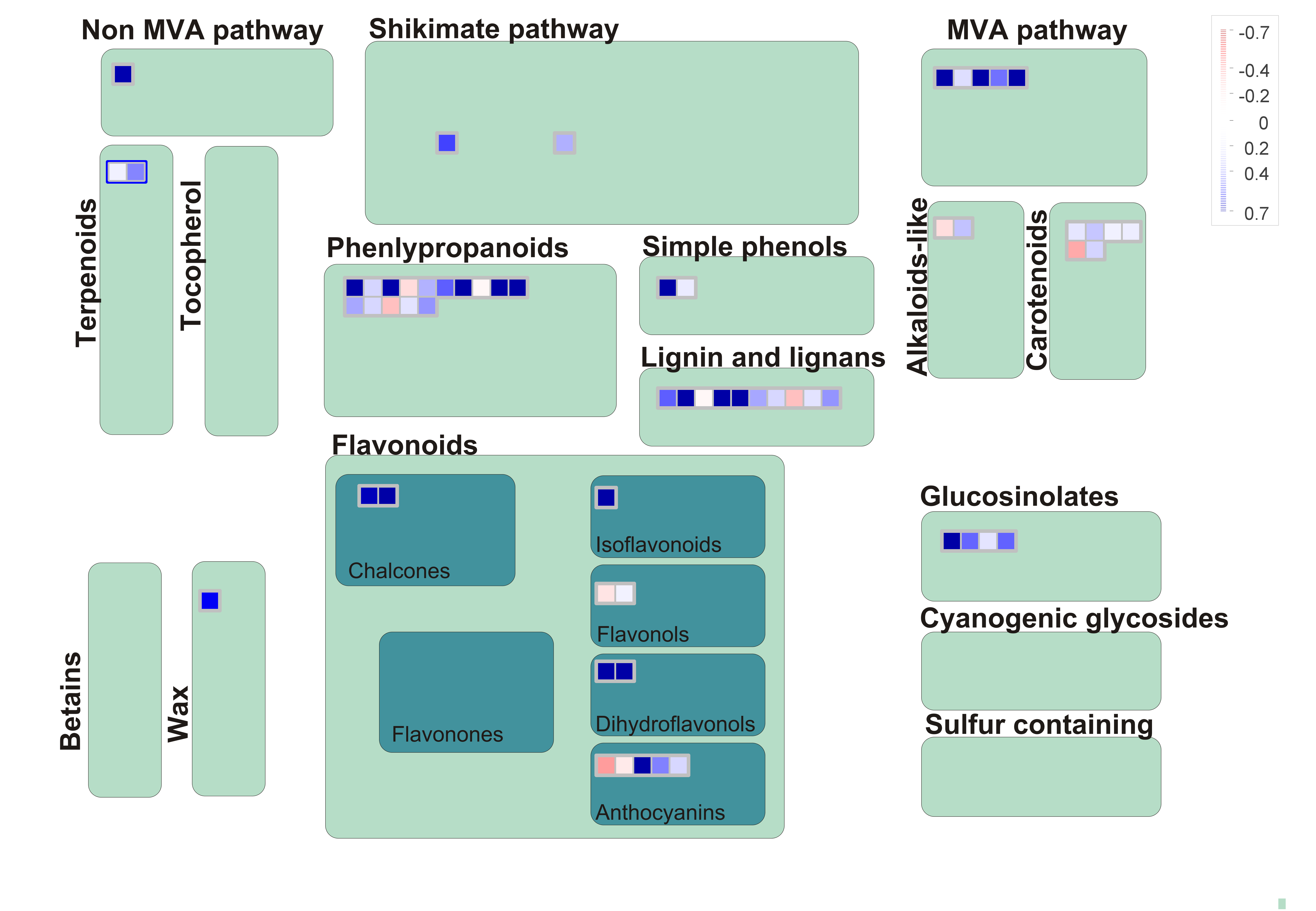

Supplement: S3 Fig — Red squares denote down-regulated transcripts and blue, upregulated. 62 data points (transcripts) mapped. Secondary metabolism is significantly up-regulated. See also Table 2 and S1 Table for details of transcripts. (PNG) [file pone.0141664.s003.png]

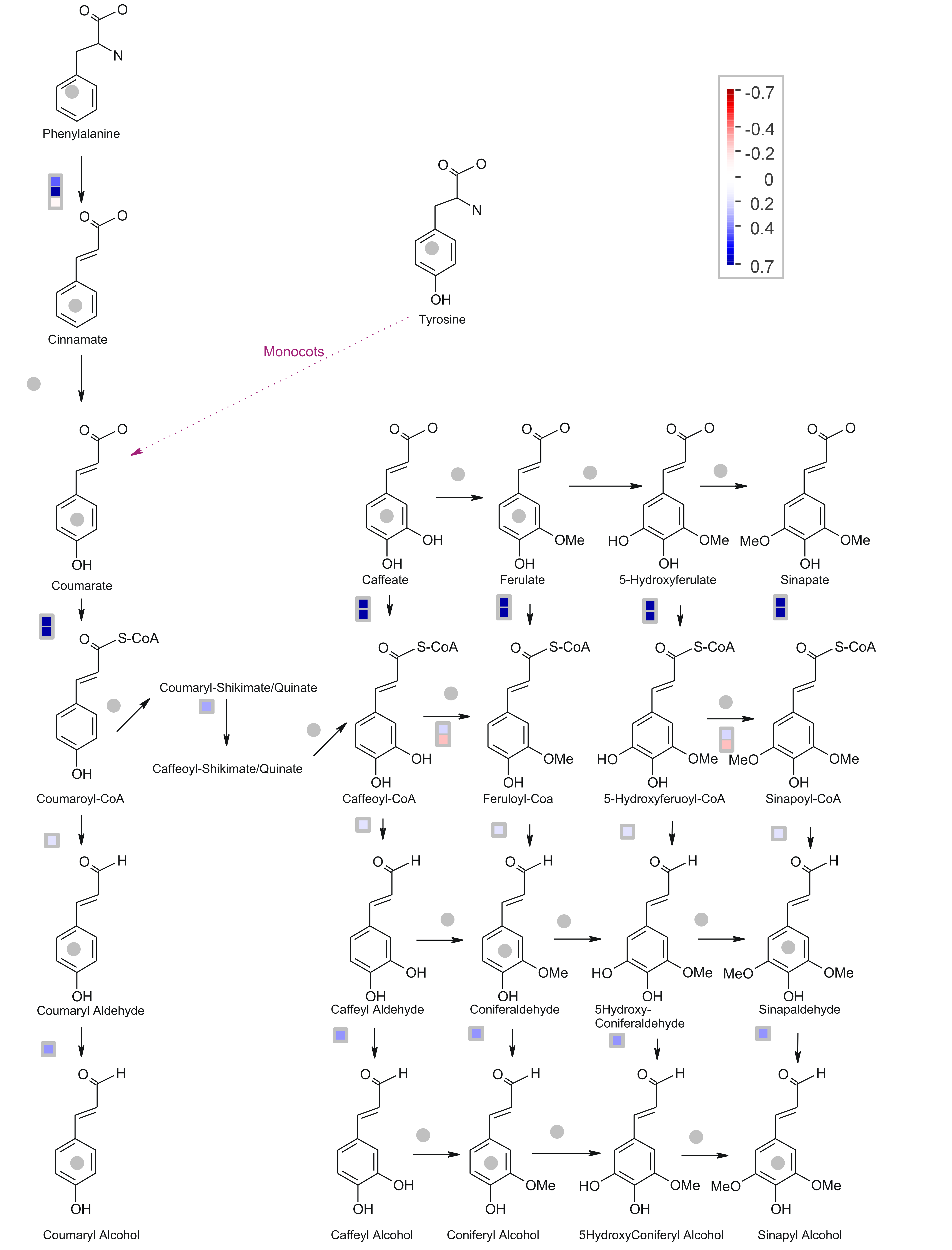

Supplement: S4 Fig — 28 data points (transcrips) are mapped and the pathway is significantly up-regulated. Red squares denote down-regulated transcripts and blue, upregulated. See text for details and S1 Table for transcript information. (PNG) [file pone.0141664.s004.png]

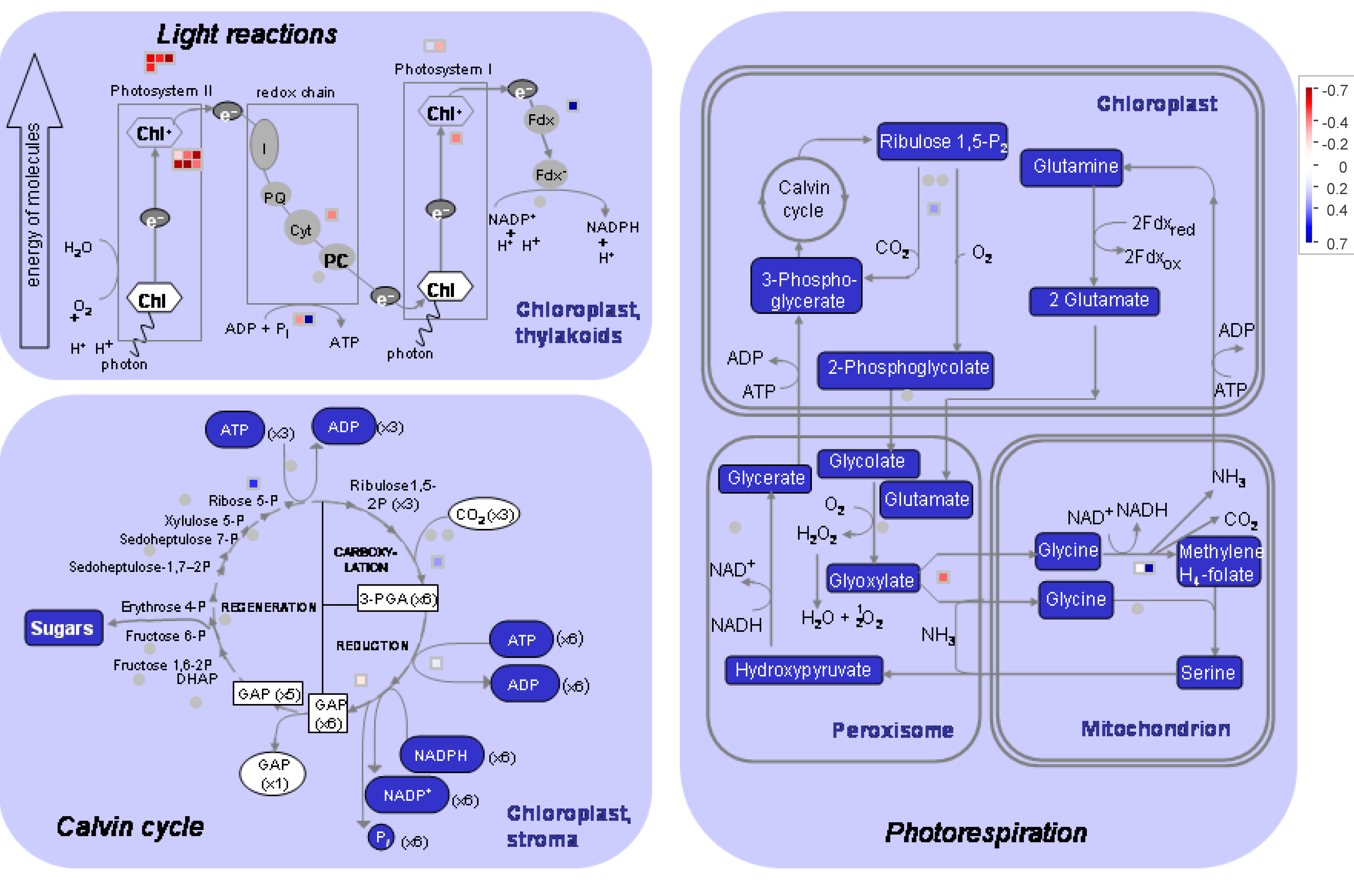

Supplement: S5 Fig — 25 data points mapped. Photosystem II pathway was down-regulated after 7 days. Red squares denote down-regulated transcripts and blue, upregulated. See paper text for details and S1 Table for transcript information. (PNG) [file pone.0141664.s005.png]

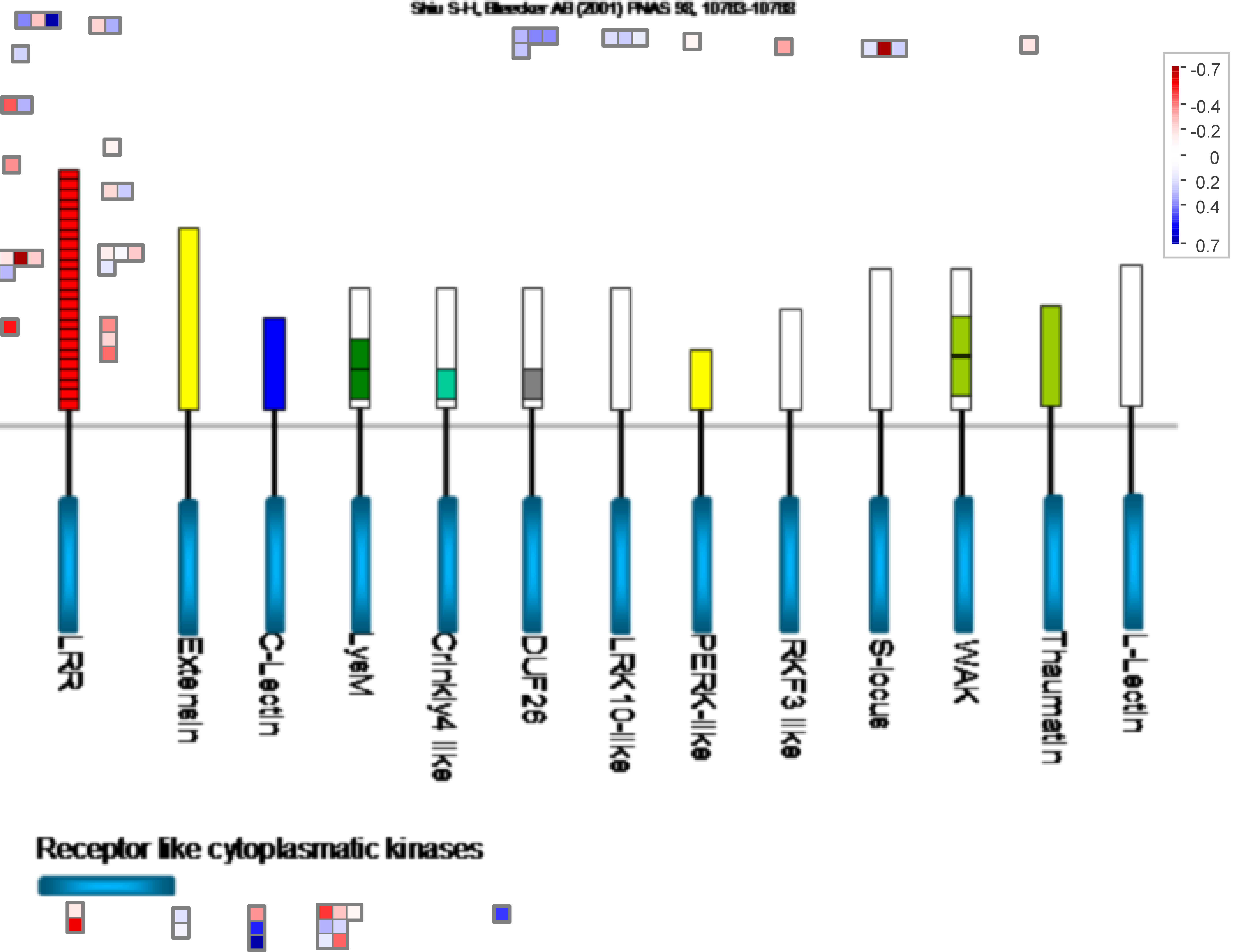

Supplement: S6 Fig — Note activity of RLK-LRR kinases (BIN 30.2) on extreme left. Red squares denote down-regulated transcripts and blue, upregulated. See text for details and S1 Table for transcript information. (PNG) [file pone.0141664.s006.png]

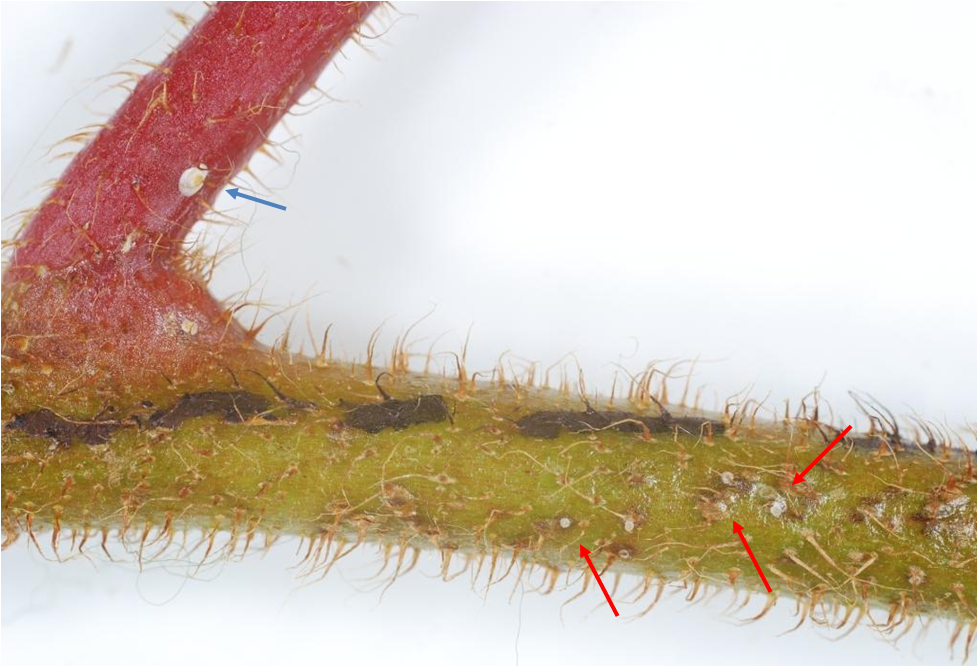

Supplement: S7 Fig — The stem is resistant to H. lataniae, but the petiole is susceptible. Note the small, dead insects on the stem (red arrows) surrounded by dead cells compared with the much larger, live insect still growing on the petiole (blue arrow). The stem is approximately 1.2cm in diameter. This could be an optimal defence strategy for a deciduous plant against a sessile pest. [black marks are marker pen]. (PNG) [file pone.0141664.s007.png]
